# Community stressors and coping mechanisms in accessing the health system during a double crisis: a qualitative case study from Yangon Region, Myanmar

**DOI:** 10.1186/s12939-023-01851-4

**Published:** 2023-03-06

**Authors:** Hnin Kalyar Kyaw, Kyu Kyu Than, Karin Diaconu, Sophie Witter

**Affiliations:** 1 Burnet Institute Myanmar & ReBUILD, Yangon, Myanmar; 2 Queen Margaret University & ReBUILD, Edinburgh, United Kingdom

**Keywords:** Health system, Health care services, COVID-19, Pandemic, Coping mechanisms

## Abstract

**Background:**

Due to the COVID-19 pandemic and political crisis, Myanmar’s health system has suspended routine services while struggling to respond to the pandemic. Many people who need continuous care, like pregnant women and people with chronic illnesses, have been facing challenges in seeking and receiving essential health services. This study explored community health seeking practices and coping mechanisms, including their views on health system stressors.

**Methods:**

This was a cross-sectional qualitative study based on 12 in-depth-interviews with pregnant people and persons with pre-existing chronic health conditions in Yangon. Sampling was purposive, convenience-based and snowball sampling was also used. The 3-delays framework was used to understand how persons were interacting with and accessing health care services; community and health system stressors and coping mechanisms in relation to COVID-19 were also identified.

**Results:**

Findings showed that Yangon region was the hardest hit with both the pandemic and political crisis and its health system was greatly affected. People were unable to access essential health services on time. The health facilities were not available to see patients, with serious shortages in human resources, medicines and equipment, resulting in interruption of essential routine services. The prices of medicines and consultation fees, and transportation costs, increased during this period. Limited options for accessing care were available due to travel restrictions and curfews. It became challenging to receive quality care because of unavailable public facilities and private hospitals being costly.

Despite these challenges, the Myanmar people and health system have shown resilience. Cohesive and organized family support structures and extended and deep social networks played an important role in accessing health care. At times of emergency, people relied on community-based social organizations for transportation and accessing essential medicines. The health system also showed resilience through establishing new service provision options, such as teleconsultations, mobile clinics, and sharing medical advice through social media.

**Conclusions:**

This is the first study in Myanmar to explore peoples’ perceptions on COVID-19, the health system and their healthcare experiences during political crisis. Although there is no easy way to cope with this dual hardship, the people and the health system, even in a fragile and shock-prone setting like Myanmar, stayed resilient by developing alternative pathways for seeking and providing health services.

## Background

Since January 2020, the world has been under attack from a new, highly contagious and fatal disease, known as COVID-19. Health systems around the world are being challenged by increasing demand for care of people with COVID-19 while trying to maintain the delivery of routine health services. When health systems are overwhelmed and people fail to access needed care, both direct mortality from an outbreak and indirect mortality from preventable and treatable conditions increase dramatically [1. –3. ].

Maintaining population trust in the capacity of the health system to safely meet essential needs and to control infection risks in health facilities is key to ensuring appropriate care-seeking behavior and adherence to public health advice. In the early phases of COVID-19 outbreak, many health systems have been able to maintain the routine health care services in addition to managing the COVID-19 burden. However, as the disease spread escalated, countries have been making difficult decisions to balance the demands of responding directly to the COVID-19 pandemic with the need to maintain the delivery of other essential health services [4. ]. In developing countries, government spending on health is relatively small. On the other hand, many developed countries are substantially financing COVID-19 related activities in their own countries, which may leave little room for providing relief funds to developing countries. Myanmar’s health sector is already challenged by common health problems faced in all developing countries, including shortage of skilled health care workers and underfunded health infrastructure. According to the World Health Organization, Myanmar is among the 57 critical human resource shortage countries, with health care workers below the WHO standard of 23 physicians, nurses, and midwives per 10,000 population. There were only 6.7 doctors, and 10 nurses and midwives per 10,000 population in Myanmar as of 2018 [5. ]. This shortage was compounded by the COVID-19 virus, which hit Myanmar’s weak health system when it first started in March 2020. The Government at that time was an elected civilian government which won the National Election in 2020 November. The civilian government was able to confine the virus with strong community coordination and national commitment [6. ].

However, with the additional military coup in February 2021, Myanmar’s health system has struggled to respond to the pandemic efficiently. Many of the routine health services have stopped [7. ]. This limited availability of the health facilities and the health care services has greatly impacted on the people, especially on the pregnant women, children and people with chronic illnesses. They are the ones who need to receive continuous and regular health care services to monitor and assess the progress and complications of pregnancy and that of diseases or treatments. The twin crisis of Myanmar has led to a great burden for them.

Yangon region is one of the most populated regions of the country, with the highest case load for COVID-19 during the pandemic. Although it has many tertiary care facilities (regional hospitals, township hospitals and urban health centres), the double crisis has distorted the system, including its health service delivery and the health care workers.

In 2021, many health care professionals followed the Civil Disobedience Movement (CDM) in opposition to the military junta and were on the streets to show their unity with the people at large [8. ]. Within the community, general practitioners and health volunteers like auxiliary midwives and community health workers have been involved in service provision for pregnant mothers and for people who need regular continuous treatment and care, yet it was not enough for those who needed facility-based care and for emergency conditions [9. ].

The study aims to provide insights into how the combination of COVID-19 and the political crisis has affected the communities which require access to services, understanding the effects of COVID-19 on service delivery at primary care levels and household coping strategies.

## Methods

This was a cross-sectional study using individual in-depth interviews with persons with preexisting health conditions. e.g., pregnant women and persons suffering from a chronic disease. The study period was from September 2020 to December 2021.

The research focused on the lived experiences of community people in accessing essential health care services when the country’s health system was starting to deteriorate with the prolonged presence of the COVID-19 pandemic and the unexpected military coup. The lived experiences of community people in seeking care would serve as a small-scale assessment of the health system and its service delivery. As we anticipated that during this horrific twin crisis, people would be unable to reach the necessary health care services, we also applied the three-delays framework to analyze collected data [10. ].

Although the three-delays framework has been principally applied to pregnancy-related emergency conditions, we believed that this concept is relevant and applicable to the other health emergencies as well. It also provides a useful framework to examine factors influencing the timeliness of care. The first delay is the delay in the decision to seek care, the second delay is related to reaching an appropriate health facility, and the third delay receiving care occurs once the patient reaches the health facility and waits to be seen by a medical professional. These delays are in turn influenced by socioeconomic factors, such as wealth and education, cultural factors such as beliefs and customs, structural factors such as accessibility of care, and health system level factors such as quality of care [11. ].

We targeted the participation of community members with pre-existing health conditions. Sampling was purposive, based in that we targeted the participation of persons known to the staff of Burnet Institute Myanmar who were 18 years old and above and willing and available for remote interviews (via phone). We additionally allowed for snowball sampling: participants were encouraged to propose other persons for participation in the study. Recruitment of participants was done via phone and all interviews were done remotely via phone. The participants were contacted prior to the interviews for their convenient date, time and medium (i.e., Viber, Messenger or phone) for interviews. For participation in interviews, given that these took up to 1-2 hours of participants’ time, the research team offered small food baskets in return for participation. They were interviewed using a semi-structured topic guide focused on exploring community stressors and coping during COVID-19. We acknowledged that participants may wish to discuss current events in Myanmar (the military coup) and how these may serve as stressors alongside COVID-19.

We interviewed 12 people via phone in the local language and at the participants’ places of choice (Table 1). Participants were categorized into medium and low socio-economic status based on their residence area, their occupation and monthly income; 5 participants were from medium socio-economic status and 7 were from low socio-economic status. 9 of them were females and 3 males. Among 9 females, 4 were pregnant mothers. Apart from those 4, all other participants had chronic illnesses such as hypertension, diabetes, a history of stroke, blood disorder and so on. The education status of these participants ranged from middle school to graduate (5 had a high level of education and 7 participants a medium level of education).

**Table 1:** Participants characteristics

Participant	Age	Sex	Socioeconomic status	Education	Disease
1	54	F	Medium	Graduate	Hyperthyroid with renal complication
2	36	F	Medium	Graduate	Post-natal lactating woman
3	73	M	Medium	Graduate	Blood disorder
4	75	F	Medium	Graduate	Diabetes, hypertension and post stroke
5	61	F	Low	7 grade	Hypertensive heart disease, diabetes
6	64	M	Low	7 grade	Chronic neuropathy
7	76	F	Low	7 grade	Hypertension, diabetes
8	38	F	Low	10 grade	Pregnant mother
9	18	F	Medium	University First year	Pregnant mother
10	60	F	Low	10 grade	Heart disease
11	61	M	Low	10grade	Hypertension, diabetes
12	33	F	Low	10 grade	Pregnant mother

Drawing on audio records from interviews, we transcribed and translated all the recordings and conducted a thematic analysis. We proceeded by inductively coding all transcribed materials and proceeded to abstract from these into categories, further synthesizing information into themes, adopting a case-based framing as relevant (i.e., considering whether salient differences exist between participants with different health conditions).

## Results

Based on salient findings, we structured the results in two large themes - the impacts of double crises on the provision of care and the impacts of double crises on accessing and using care. The sub-themes under the impacts of double crises on the provision of care were: availability of services at the health facilities, availability of service providers at the health facilities and availability of medicines, equipment and other commodities. The sub-themes under the impacts of double crises on accessing and using care were: seeking care, reaching care and receiving quality care (according to three-delay framework). Lastly, we also explored how people cope with the crisis, including how they received information to cope with the crisis and sought available alternative health care services.

## The impacts of double crises on the provision of care

Although Myanmar responded early to the COVID-19 breakout, it faced three major waves 1 of the pandemic, starting from March 2020. The findings here will be described wave by wave.

During the first wave, from March to August 2020, there were only 374 cases and six deaths. The second wave started on 16 August in Rakhine State, after almost a month without local transmissions. Myanmar saw a dramatic increase in the second wave (August 2020 to February 2021) to around 1,000 new cases daily.^1^ This dramatic rise was overshot in the third wave (June-September 2021) which saw around 5,000 daily new confirmed cases. By the end of the third wave in September 2021 there were 462,608 cases with 17,682 deaths.

### Availability of services at the health facilities

It was found that the health facilities which had been opened and easily accessible were not available anymore during the COVID-19 period. During the first and second waves of COVID-19, almost all the health facilities and health staff shifted their efforts to COVID-19 prevention and care activities rather than routine health care services.

According to the participants, this shift caused interruption to the routine health services such as antenatal care, immunization, NCD management and basic emergency care in most of the public health facilities. This limited availability reached its peak in the third wave. During the third wave of COVID-19, the overburdened Myanmar health system had to face the additional crisis of serious human resource shortages and challenges of personal safety from the unexpected political situation, which has made it a lot more difficult for the service users to access routine essential health care services.

*“Yes … this pregnancy is … much more difficult for me. In previous pregnancies, I could easily access to the hospitals. And I did not have to worry about receiving AN care at the UHC (urban health centre), receiving the blood testing and urine testing services.” (IDI 8)*

Pregnant women faced difficulties in seeking routine obstetric care services because many primary care facilities within their reach that had previously provided these services, shut down. To receive those services, some had to travel for a very long distance and had to spend much more time to get to the services.

*“Since the health centres near me are all closed, they told me to go to the Gymkhana (the largest tertiary hospital for Women’s Health in Myanmar, also known as Central Women's Hospital) which was very far from South Dagon (about an hour from my home with a taxi) I had to go there four times to get one antenatal service … and it costed a lot each time … I left …. in the morning …. around five am in the morning and arrived at the hospital around seven am.” (IDI 8)*

Some people with chronic diseases needed to change their service usage from public hospital to private hospital as public hospitals were not able to provide the usual services during the COVID-19 period.

*“We could not go to North Okkala General Hospital since February. All the public hospitals were not able to give regular services including blood transfusion since February. So, we had to change to private hospitals to get blood transfusion, which was very costly” (IDI 3)*

Moreover, COVID-19 positive patients were not able to receive services in government public hospitals as there was a severe imbalance between the patient load and health staff. As many of the COVID-19 positive cases were not treated in time, community spread worsened and demand for health services was intense, especially during 3^rd^ wave, and home-based care for COVID-19 was the only option, involving a terrible chase for oxygen supply by the community.

*“It was … my next-door neighbour. ….. He died. He went to Yangon General Hospital and no space available there, so he went to North Okkala General Hospital because his oxygen level kept dropping. He couldn’t get a chance to be admitted at the hospital and came back home. He died immediately after coming back home. That’s what happened near my home. This is the reality.” (IDI 11)*

Additionally, the services at the general practitioner clinics in the community reduced compared to the first and second waves. Many of the clinics shut down due to the high intensity of community spread of COVID-19 infection in the third wave.

*“It was …. sometimes, during this period, naturally, .. the doctors were afraid of … they sprayed with sanitizers when patients left their clinics and closed the clinic .. many difficulties… So, we had to inquire which clinic opened because many clinics closed at that time and doctors are also afraid of accepting the COVID-19 patients.” (IDI 11)*

### Availability of service providers at the health care facilities

The primary, secondary and tertiary care facilities faced serious shortages of skilled health staff not only because of COVID-19 infections hitting health staff but also because many health staff were in the Civil Disobedience Movement, which was a peaceful resistance by the civil servants to the military coup, during the third wave.

*“The problem was doctors and nurses’ shortage. I felt so sorry for the doctors and nurses at the hospital. There were many patients and only a very few doctors, they had huge workload, they must be very tired. As a result, they became less efficient. I was punctured 3 or 4 times for one blood test.” (IDI 3)*

Patients outnumbered the capacity of the health staff in the health facilities, especially at the tertiary health care facilities.

*“So, with lesser number of skillful professionals, it is harder for the patients. And there were situations like patients outnumbered the health staff. It was such a hectic situation.” (IDI 3)*

In the community, the people could still rely on the services provided by the community providers and health volunteers like auxiliary midwives and community health workers for minor illnesses.

*“If I encounter health problems, … I will ask help from Sayarma (an auxiliary midwife). I have only her near me to rely on. She said not to worry about the delivery. She said she would be there for me. So, I rely on her.” (IDI 9)*

### Availability of medicines, equipment and other commodities

With increasing prices during the COVID-19 period, people faced serious shortages of drugs, especially in the third wave. This impact was significantly seen on the COVID-19 positive patients, and on the people with chronic illnesses, especially during the peak of the outbreak.

*“Because at that time, we could not buy the usual brand and only the similar ones were available. The last time, it was … the stocks were very limited, even paracetamol was not available. I always bought paracetamol India brand with 200 Kyats for one strip, but it became 1,500 for one strip. The pain killers were all stocked out. The demand was so high. Thus, the price became very much higher.” (IDI 6)*

Compared to first and second waves of COVID-19, the tertiary health facilities have been facing shortages of essential drugs and other medical supplies most seriously during the third wave. The most important and significant shortage was the unavailability of oxygen supplies for COVID-19 patients, which took many lives.

*“He died [the neighbor] due to not getting oxygen in time. Although he went to two big hospitals. Oxygen was so scarce, and the only hope was at the hospitals and so sad even though he reached there… he did not receive it. (IDI 11)*

## The impacts of double crises on accessing and using care

We structured our findings on utilization of care according to the three-delay model.

### Seeking care

Regarding care seeking practices, almost all pregnant women who participated in the study had knowledge and previous experiences of when to seek health care relating to pregnancy and delivery. However, it differed in the chronic care group. These people often lacked knowledge of regular and emergency care, depending upon the duration of diagnosis. For those with longer illnesses, they often thought that it could be treated by themselves using simple remedies. This perception may put them into danger because of delays in seeking care.

Seeking care also depends on financial flexibility. Those of medium socio-economic status were abler to overcome the obstacles in seeking health care and to plan for emergency situations than those of low socioeconomic status.

For the people with a low socio-economic status, during the COVID-19 third wave where the twin crises happened, financial difficulty became the major challenge in seeking necessary skilled obstetric and other medical care. Due to their inability to access skilled care in this phase, people with low socio-economic status reported feelings of hopelessness, which could cause negative pregnancy and disease outcomes.

*“Right now, my husband’s work is not very good because there was long period of closing down, his job was suspended for two months. I have no job now. Previously, me and my mother opened a small shop of “Mont-hin-khar (Myanmar traditional rice noodle)”, but now, we have to close the shop as people were afraid to buy and eat from outside vendors and it is very difficult for us. I haven’t prepared anything for the delivery yet.” (IDI 12)*

*“This time I got pregnant, I felt really depressed and even thought about …. nonsense … like… due to these COVID-19 and coup crises … I even thought about having an abortion. But I controlled my mind and took care of this pregnancy since it is reaching to its fourth or fifth month. I have been depressing a lot. (IDI 8)*

There was reticence in some cases of low socio-economic status individuals seeking care due to COVID-19 due to concerns about the burden it may impose on the family.

*“I told my family that … I will try to take care of myself as much as possible. But if something happens, and I need to be hospitalized, I would rather die. I don’t want to give troubles to anyone, no one is financially ok. It would be like giving my burden to the others.” (IDI 10)*

Regarding knowledge of the disease process, people with medium socio-economic status (and often education levels) seemed to better understand their disease and care process and to have more knowledge on when to seek care.

*“Although, my doctors asked me to check-up with them every two to three months, I regularly did my lab tests once a month just to monitor myself.” (IDI 1)*

### Reaching care

During the COVID-19 period, it was very hard for the community to reach care. Transportation difficulties were one of the major problems described by the participants in reaching the health care facilities, especially for the pregnant women of lower socio-economic status. Many of the participants needed to spend larger amounts of money per month, e.g. approximately one-third of the monthly earning for some people, on the transportation cost per visit as the only available health facility was so far from their residence. For emergency conditions and at night, many philanthropic organizations helped the community by providing ambulance services, yet this was of limited help for those who seek regular care.

*“For one visit, transportation alone costed me 15,000 MMK. I had to leave home very early in the morning around 5 am to reach there in time at 7 am.” (IDI 8)*

Transportation cost was not a serious problem for the pregnant women of medium socioeconomic status. But these participants also needed to worry and plan for emergency conditions, since night-time curfews had been announced in almost all townships of Yangon (this was stricter during the third wave of COVID-19).

*“At that time, there was a curfew … and I was thinking of how I would get to the hospital if there was an emergency … how to do if I needed an ambulance … who would come with us…. and so on. We live in Sanchanung, so I gathered all the information of the social support organizations in Sanchaung and noted down and contacted them in advance. That was quite an amount of work.” (IDI 2)*

For people with chronic illnesses, reaching a health facility even in normal times was a different experience for people of different socioeconomic status. According to the study participants in the medium socio-economic group, more options such as private clinics and specialist care were available as alternatives to health care services at the public health facilities. For those in the lower socio-economic group, fewer options were available and accessible.

*“I could not afford to consult with a specialist doctor since I have been struggling to earn money for daily foods. Providing food for the family daily was a bigger struggle for me. I asked the doctor … if I could not come anymore, would it be possible for me to continue the prescribed drugs on my own. He said to continue taking the prescribed drugs if I could not come for regular follow-up. If I have severe shortness of breath and chest pain, I go to the general practitioner near here and took some injections.” (IDI 10)*

No matter what their socio-economic status is, it was evident that people needed to spend more time and money than usual in reaching care, especially during the third wave. Thus, when they did not have sufficient family support, people could not reach health care in time. It was found that most of the people from lower socio-economic groups did not intend to ask their family members for help and support since they did not want to put more burden on the family members who had been struggling for daily needs and food. Thus, some of them said they would rather die than go to the health care service, the cost of which they would not be able to afford during this difficult time. Additionally, the limited availability of physical cash in-hand was one of the major reasons stopping people accessing health care. This started just after the February 2021 political crisis; many of the banks started to limit daily cash limits from the ATM machines as people rushed to withdraw to buy essential goods for their families. This bank crisis resulted in physical cash scarcity for the ordinary people.

*“For us, we had just enough money to stay alive. It was so much worse than before. If there would be an emergency now, … I mean an emergency health issue which would cost us 5 or 10 million, we would rather die.” (IDI 11)*

*“Another issue is the cash. We dare not spend the cash we have in hand. As you know, physical cash is very scarce now, and cash cannot be taken out easily from any bank since the coup. We need to save it. Example – if you feel ill .. at some hospital, you know … when we go to Grand Hanthar (private tertiary hospital), they accept to be paid with the card or mobile banking, but that is not available after every 20th of the month. Shwe Lamin (private hospital) doesn’t accept mobile banking at all. We need to pay for everything in cash. That is why I made it clear at home, if one of you wants to go and get the services at the private hospitals, go before the 20th. (laughing)” (IDI 1)*

All participants, rich or poor and with or without family support, mentioned that they did not really have solid plans for an emergency. Even the people from medium socio-economic status were unsure what to do, who to turn to and to which health facility they would go because there were very limited health resources around the city during the twin crisis.

*“I … right now … really … think … I think that I am helpless. I don’t know where to go if something happens, you know. The emergency unit at Yangon General Hospital is …. How can I say? … I am not sure if it is still operating now due to CDM and Non-CDM issues. Another news I heard was …. If COVID-19 positive patients come in, I heard that they refused to admit, they refused to accept these patients.” (IDI 1)*

### Receiving quality care

Regarding receiving quality care, some people needed to move from the public government hospital to the private hospitals to continue regular treatment for their illness, which cost much more than in the government hospital, which became a burden for the patients.

*“We could not go to public hospitals since February, so we went to private hospitals for blood transfusion. Example - if I can go to the North Okkala government hospital for blood transfusion, it would cost much lesser. And because of COVID-19 risk, the expenses become much higher at the private hospital.” (IDI 3)*

Receiving quality care at the public or private hospitals became very challenging for all people, no matter what their socio-economic status is, especially after the military coup in February.

*“So, comparing these 2 periods, in the first period, people only suffered from the COVID-19 impact because the government was good; but in second period, people have additional burdens besides COVID-19 because the government was bad. As you know, many people have faced the life-threatening situations more than before. The worst thing they did was limiting the medical equipment and drugs, and finally arresting the doctors and nurses, restricting the activities of philanthropic organizations.” (IDI 3)*

While people could not reach care in time during this period, many physicians and consultants provided teleconsultation services Physicians were seeing patients from their home as they were also afraid of the disease and did clinical consultations through private clinics via Zoom, Viber and Messenger services. However, the service charges were the same as seeing a patient in person and the consultation fees were considered to be one of the challenges in receiving quality care, because during the COVID-19 period people had fewer job opportunities, resulting in lower earnings than before.

*“At that time, my renal doctor had stopped practicing at the clinics completely because he followed the civil disobedient movement [peaceful resistance by the civil servants against the military takeover] and only the teleconsultation or the messenger was available to be in touch with him.” (IDI 1)*

*“And the consultant fees have been starting to increase since that time. Previously, my wife went to the clinic near the post office which costed only 4,000 – 5,000 each time. Now, it increased to 9,000.” (IDI 6)*

## How households coped with the double crises

In our study, we found that many people coped with the twin crises of COVID-19 and the political unrest by relying on their faith and religious teachings. Family support structures also played an important role in helping families to cope. Many of our study participants mentioned that they could overcome the stresses and challenges with the support from their family members. We also found that having a good social network was very important to overcome the obstacles faced. Apart from good family support structure and good social network, study participants stated that having small children in the family was one of their stress relievers. Regardless of the socio-economic status, it was noticeable that if participant had a good and organized family support structure, they were better able to access health care more.

*“My son is very supportive and is like … I don’t even need to say a word. My daughter-in-law is also very good to me, so is my son-in-law. They said, “the money didn’t matter, only mom’s health mattered.” My daughter-in-law, son and younger daughter, all of them took great care of me.” (IDI 4)*

Regarding COVID-19 restriction measures, it was found in our study that people followed the rules and restrictions that were announced by the Ministry of Health very well. At first, people found it hard to follow these restrictions, but the fear of contracting the infection made them accept and follow the rules. To cope with COVID-19 situation, study participants reported that they had changed their lifestyle by wearing masks, washing hands frequently, and practicing the social distancing effectively.

*“I think people follow these measures seriously. Because people get frightened since there are many deaths. No one dared to go out. The streets were cleared. Because they were afraid. Afraid to get the infection.” (IDI 6)*

Our study participants tried to find alternative pathways to receive the necessary health care services, such as teleconsultations and mobile clinics. The community-based philanthropic organizations and community-based health volunteers played very important roles in households seeking care practices during this difficult time of COVID-19. They supported community members in searching for oxygen and necessary and scarce medicines for COVID-19, but faced official restrictions on their own activities.

*“And to fight COVID-19, ….. with the previous government, many philanthropies … like U (name) and his foundation provided lands, buildings, and donated millions of money to the government to fight COVID-19. Many people were supported and that is why I think there were lesser positive cases back then. But now, the philanthropies dare not donate oxygen and other necessary things widely in public. Our current motto is “From the people to the people”. So, some active people tried to stabilize the prices of foods and other goods by selling with much lower prices. But they cannot do that very widely because the military tries to arrest them very often.” (IDI 3)*

Regarding receiving information to cope with the COVID-19 situation, almost all of the participants reported receiving updated information from Facebook as a primary source. After the coup, people mainly relied on Facebook for information and updated news, rather than state-owned TV channels and newspapers, yet a few of study participants tried to receive news and information from the newspapers. Almost all participants thought that TV channels were not a reliable source of information during this twin crisis. People’s trust in the news media has changed since the February coup.

*“On TV …. The news is …. The news could be biased because of the political reasons.*

*.. so, I think it is not that reliable.” (IDI 9)*

*“Previously, regarding the information, we relied on the government’s broadcasting channels, example – previously with Aunty Su’s government [elected civil government], she personally broadcasted the information on TV to get people's attention more. I think this way, the message and information that government wanted to give reached more to the people. Now, we do not watch TV news, not listen to radio news anymore, frankly speaking, it is because they announced many fake news.” (IDI 3)*

During the first and second waves of COVID-19, before the coup, people relied on the Ministry of Health and Sports Facebook page very much for all the COVID-19 news, information, and preventive measures, but this changed after the coup.

*“Previously, when you look at the MOHS Facebook page, you know how many new cases, increasing or decreasing, you know it exactly. Now, it is not good anymore, you cannot get this information from MOHS page plus I don’t watch TV anymore.” (IDI 1)*

*“Previously, we watched TV, listened to the advises from the respectable professionals, we received the information this way. But now, we have to rely on the advises posted on Facebook by the doctors, professionals, respectable volunteers and technical persons. … We mainly get the information from the trustworthy and respectable online news media.” (IDI 3)*

Regarding receiving information for available alternative health care services like teleconsultation services for routine care and treatment, online COVID-19 consultation services for positive patients and information on the availability of oxygen, medicines and other necessary medical equipment, these were all updated mainly on Facebook. People needed to check the respective Facebook pages every day to receive updated information during all three waves of COVID-19.

*“I feel like I have enough knowledge. Like I said .. many people shared on Facebook if there is something new. I use it most of the time, so I think am up to date. I don’t watch TV but Facebook and You tube. I tried to get the updated news as much as I can.” (IDI 2)*

*“I read and followed the posts on Facebook posted by the well-known physicians.” (IDI 11)*

## Discussion

In this study we identified that both COVID-19 and the ongoing political crisis in Myanmar had substantive effects on the health care system, resulting in disruption of services. According to users, health care facilities were overwhelmed by COVID-19 patient numbers, fewer staff were available to offer health services and availability of key commodities and medications was also compromised; in many cases health facilities had to close. This was consistent with the findings from other studies with similar settings. Ahmed et al described that in Bangladesh, with the imposition of restrictions due to COVID-19, there was disruption to health care services. Some no longer functioning at all [12. ]. Not only in the same region, but also in other parts of the world, the services operated with reduced health staff and the operation of many facilities had been suspended, according to Giannopoulou et al. [13. ].

We identified that care seeking was disrupted for all interviewed patients, however this took different form for members of different socio-economic strata. Those most vulnerable in society – who could not afford care seeking at private facilities – needed to seek care at public facilities which in cases were closed. For those of medium-socio economic status, or with good family ties that were able to step in and support care seeking, disruptions were also noted but fewer, as care seeking at private facilities could still be arranged under specific conditions. However, economic conditions deteriorated significantly in the country, making many patients aware that seeking care would potentially be a burden on the wider family. This particularly increased in third stage of the pandemic. A similar finding was seen in a study conducted in slum communities of Bangladesh, Kenya, Nigeria and Pakistan, which highlighted the impact of COVID-19 restriction measures on daily labourers [12. ]. The laying off of many workers due to COVID-19 restrictions and the impending economic crisis have cause an overall burden to the family [13. ]. This may impact maternal and newborn nutrition, and thus pregnancy outcomes and child stunting in future, especially for vulnerable populations [14. , 15. ].

Evolving challenges were also evident for community members attempting to seek care. For the first delay, families went from fear of infection in the first and second waves of COVID-19 to inaccessible services in the third wave and reduced trust in the system. For the second delay, rise in costs, transport challenges, alongside reduced economic means for the households, were the main barriers. For the third delay, increased service costs, reduced availability and concerns about burdening their household were significant, all of which increased in the third wave. In our study, it was found that many community-based organizations helped people in seeking care by transferring patients to hospitals with the provision of first aid and ambulance services.

According to Polizzi et al, the COVID-19 pandemic led to feelings of helplessness and the loss of a fundamental sense of safety, security, financial stability, and the ability to envision a brighter future. Fear of infection in the presence of others, of contact with contaminated surfaces, and of passing too close to another human being evoked a shudder of mistrust of others, avoidance, and withdrawal from everyday activities [16. ]. These kinds of responses and feelings were found among our study participants. Our study participant experienced feeling unsafe and insecure, anxious from financial instability and by practicing ‘social distancing’, they withdrew from social interactions and activities. Taking part in social activities and seeking comfort from others are the usual ways of reducing stress, which were not possible during COVID-19. Reaching out to others via modern technology and social media, supporting others via expressing empathy, active listening, sharing resources, and demonstrating consideration by adhering to the “rules” of social distancing are ways of managing this problem [16. ]. Our study participants also followed these practices and contacted their loved ones via social media. Polizzi et al also stated that finding ways to engage with and appreciate life during mass traumas was a robust predictor of increased psychological well-being and reduced post-traumatic stress symptoms. These sorts of coping activities, called behavioural activation, are diverting and spark positive emotions that researchers found to be critical to resilient outcomes and recovery [16. ].

While the communities in Myanmar tried to cope with the crisis in their own way, the health staff, mainly the staff who followed the Civil Disobedience Movement, showed their resilience by providing online consultation and counselling services, especially during the third wave of COVID-19. In the months to come, we could cultivate health system resilience by incorporating innovative models of digital health care as an alternative pathway [17. –20. ].

This is the first study in Myanmar to explore peoples’ perceptions of COVID-19, the health system and their healthcare experiences during double crises. In particular, our study highlighted the challenges of coping with a double crisis – COVID-19 on its own was being managed effectively prior to the coup, but the combination of political crisis with epidemiological one caused major hardship, especially for less resourced and connected patients. It also highlighted the important insights into resilience and coping strategies, including the importance of community-based organizations and volunteers and social networks, especially in times of crisis. Moreover, the potential role of social media in combatting the COVID-19 pandemic was also highlighted in our study. In Myanmar, the major social media platform that people used was Facebook. Myanmar’s people, including our study participants, shared positives messages and updated news or information on the COVID-19 situation, do’s and don’ts on Facebook accepting that ‘sharing is caring’, while many other people reduced their stress by engaging in a variety of hobbies and mentally challenging tasks.

The study was affected by the political instability, which led to a reduced scope and also scale of data collection (with a limited number of in-depth interviews). The study was only conducted in Yangon. Recruitment of participants was by purposive sampling and snowball sampling methods which could bias participant selection. The research findings do not represent the whole of Myanmar population and there are likely to be different challenges for people living in rural areas. Since this study explored health system stressors indirectly, further work involving in-depth exploration of health care worker perspectives is recommended. The study could also be expanded to other areas and populations.

## Conclusion

The COVID-19 pandemic had traumatic effects on community members, both directly and in terms of its effects on an already fragile health system. Although the overburdened Myanmar health system was further damaged by the effects of the military coup, it showed its resilience by finding alternative pathways to provide services. The limited resources and gaps in the health system exposed during the COVID-19 outbreak gave us an opportunity to reconsider how services should be organized and delivered, including through potentially expanding the telemedicine system to reach those in remote areas, where internet coverage has been growing. For community members, family support structures and community networks were key to resilience in the difficult double crisis period.

## Data Availability

The dataset used and/or analysed are available from the corresponding author on reasonable request.

## Footnotes

1 https://covid19.who.int/region/searo/country/mm
